# Salivary Scca1, Scca2 and Trop2 in Oral Cancer Patients—A Cross-Sectional Pilot Study

**DOI:** 10.3390/dj10040070

**Published:** 2022-04-15

**Authors:** Ivana Karmelić, Ivan Salarić, Ksenija Baždarić, Marko Rožman, Ivan Zajc, Marinka Mravak-Stipetić, Ivona Bago, Davor Brajdić, Jasna Lovrić, Darko Macan

**Affiliations:** 1 Department of Medical Chemistry, Biochemistry and Clinical Chemistry, University of Zagreb School of Medicine, 10000 Zagreb, Croatia; ivana.karmelic@mef.hr (I.K.); zajc@sfzg.hr (I.Z.); jasna.lovric@mef.hr (J.L.); 2 Department of Oral Surgery, University of Zagreb School of Dental Medicine, Av. Gojka Šuška 6, 10000 Zagreb, Croatia; dbrajdic@kbd.hr; 3 Department of Maxillofacial and Oral Surgery, University Hospital Dubrava, 10000 Zagreb, Croatia; 4 Department of Medical Informatics, Faculty of Medicine, University of Rijeka, 51000 Rijeka, Croatia; ksenija.bazdaric@medri.uniri.hr; 5 Department of Physical Chemistry, Ruđer Bošković Institute, 10000 Zagreb, Croatia; marko.rozman@irb.hr; 6 Department of Oral Medicine, University of Zagreb School of Dental Medicine, 10000 Zagreb, Croatia; mravak@sfzg.hr; 7 Department of Endodontics and Restorative Dentistry, University of Zagreb School of Dental Medicine, 10000 Zagreb, Croatia; bago@sfzg.hr

**Keywords:** oral cancer, early diagnosis, biomarkers, saliva

## Abstract

Oral squamous cell carcinoma (OSCC) is frequently diagnosed in the advanced stages. The purpose of this paper is to determine the salivary values of SCCA1, SCCA2 and TROP2 in patients with T1N0M0 OSCC and to compare them with the values obtained from healthy individuals. Unstimulated (UWS) and stimulated (SWS) saliva was sampled from 29 patients with T1N0M0 OSCC and 29 sex- and age-matched healthy individuals. Statistical difference was observed in SCCA1 and SCCA2 levels both in UWS and SWS samples. TROP2 was not measurable in most of the salivary samples. Both SCCA1 and SCCA2 could represent potential biomarkers for the early-stage OSCC. Research on a larger sample and biomarker validation is needed to assess the clinical potential of SCCA1 and SCCA2 in the OSCC early diagnostics.

## 1. Introduction

As reported by GLOBOCAN, oral cavity and lip cancer had an incidence of 377,713 cases, with 177,757 deaths from this disease in 2020 [1]. Although lip and oral cavity cancers are most often squamous cell carcinoma types, they do not share the same etiology. Despite the easy-accessible examination, almost 60% of all oral cavity squamous cell carcinomas (OSCC) are diagnosed when metastatic disease is already present [2,3]. The most commonly associated risk habits with this malignancy are tobacco and alcohol consumption [4].

The search for the early-stage OSCC biomarker has been a goal for a number of studies, however, none of them yielded with a clinically exploitable biomarker or biomarkers for OSCC early diagnosis.

Squamous cell carcinoma antigen (SCCA) has been described in the literature as a diagnostic biomarker for squamous cell carcinomas of the uterine, cervix, hepatocellular, esophagus, lung, uterine cervix, head and neck cancer and colon [5,6,7,8] with the possibility of reflecting tumour stage, size, stromal, lymph node and vessel invasion and overall prognosis. Two genes, located at the 18q21.3, encode the two isoforms of SCCA [9]. SCCA proteins belong to the serine proteinase inhibitor family. SCCA 1 (SERPIN B3), the neutral isoform, inhibits the cysteine proteinases cathepsin K, L and S, while SCCA2 (SERPIN B4), the acidic isoform, inhibits the chymotrypsin-like proteinases, cathepsin G and mast cell chymase. Both SCCA1 and SCCA2 are present in the stratified squamous epithelium of healthy individuals and in squamous cell carcinomas, but not in the circulation of healthy individuals [7]. Mainly, SCCA2 is measurable and detected in the serum of patients with squamous cell carcinomas [10]. Several studies recognized serum SCCA as a potential biomarker for oral cancer diagnosis, alone or as a part of a diagnostic panel with other molecules [6,10,11]. The downside of serum SCCA1 and SCCA 2 as biomarkers in OSCC diagnosis is the apparent lack of specificity.

Human trophoblast cell-surface antigen (TROP2), also known as TACSTD2/GA733-1, M1S1 or EGP-1, encoded by the TROP2 (also known as TACSTD2) gene on 1p32 chromosome, is a transmembrane glycoprotein rarely expressed in healthy tissues, and its role, although not yet clear, is believed to be linked with tumour development and tumour growth regulation [12]. Several studies demonstrated the potential of TROP2 as a diagnostic or prognostic biomarker [13,14,15].

A study was carried out by Fong et al. on oral squamous cell patients, where in 58% of tumour samples, TROP2 overexpression occurred [12]. Although investigated in many malignancies, the role and molecular mechanisms of TROP2 in OSCC is still unknown, but it is believed that when the expression of this protein is high, TROP2 has oncogenic properties and induces the tumour adhesion, migration and invasion.

As a transmembrane glycoprotein, it is interesting that it was measurable and elevated in the serum samples of patients with non-small-cell lung cancer compared to healthy patients [15].

Saliva is a body fluid which, due to its proximity, accessibility and greater cost-effectiveness compared to blood sampling, is the target of many biomarker research studies. The proximity to OSCC has made it particularly interesting in the OSCC biomarker research. Unfortunately, a number of studies revealed only potential salivary biomarkers for OSCC and almost all require validation with a greater sample. None of the potential salivary biomarkers for OSCC described in the literature showed satisfactory sensitivity and specificity.

The aim of this study is to measure salivary SCCA1, SCCA2 and TROP2 levels in patients with early-stage T1N0M0 OSCC. To the best of our knowledge, SCCA1, SCCA2 or TROP2 have not been measured in the saliva of oral cancer patients.

## 2. Materials and Methods

STROBE recommendations have been followed for this manuscript. The methodology described in this study is congruent with the one by Salarić et al. [16].

Only patients with verified T1N0M0 OSCC (in correspondence with the 8th edition of the American Joint Cancer Committee [17]) were included in this study. Furthermore, only OSCC patients that had no history of previous oral malignancy were included in this study. Patients with tongue root and epiglottis OSCC were not included. Sex- and age-matched respondents that came for examination or a tooth extraction were included as a control group. No invasive procedure was carried out prior to saliva sampling in both groups. Patients with OSCC were sampled the morning before surgery.

Inclusion criteria for the OSCC and control group were [16]:

- Not eating or drinking at least 8 h before sampling
- Not brushing teeth or using a mouthwash at least 1 h before sampling
- No salivary, jaw and oral mucosal tissue diseases or conditions (apart from gingivitis and periodontitis)
- No history of radiation therapy of the head and neck

Saliva sampling was carried out from November 2018 till June 2021 and performed at least three weeks after the biopsy was taken. All samples were obtained under the same conditions (between 7 and 9 a.m.), and both unstimulated (UWS) and stimulated whole saliva (SWS) were sampled using a specially designed saliva collection apparatus [16]. Saliva was stimulated by chewing the paraffin blocks. Samples were handled as described by Salarić et al. [16]: stored at −80 °C within 30 min after sampling and until Enzyme-Linked Immuno-Sorbent Assay (ELISA) testing. Instructions of the ELISA kit manufacturers (For SCCA1 and SCCA2: MyBioSource Inc., San Diego, CA, USA; for TROP2: Elabscience Biotechnology Inc., Houston, TX, USA) were strictly followed. Saliva samples were centrifuged for 20 min at 1000× *g* according to manufacturer’s instructions. Afterwards, we removed the particulates and stored the samples in aliquots at −80 °C. We avoided repeated freeze and thaw cycles. Optimal sample dilutions were separately determined for all three biomarkers. Preliminary experiments to determine the validity of the kits were carried out as were the preliminary experiments before the general assay for each batch. The lower limit of detection was determined by adding two standard deviations to the mean optical density value of twenty zero standard replicates and by calculating the corresponding concentration for all three biomarkers. For SCCA1 preliminary test revealed low concentrations of SCCA1 in the saliva sample, so control samples (serum and plasma) were used to control the ELISA kit. The linearity of saliva measurements was initially tested on four samples in four different dilutions (undiluted sample, 1:1, 1:5 and 1:10) (Supplement 1). For SCCA1 and SCCA2, analysis was performed in duplicate for each sample and the controls.

Medical and dental history were taken for each respondent. Drug consumption was grouped using Anatomical Therapeutic Chemical Classification System (ATC) [18] and the systemic diseases by the International Statistical Classification of Diseases and Related Health Problems 11 (ICD-11) [19]. Alcohol and tobacco consumption was expressed as alcohol units (a.u.) and average number of cigarettes smoked per day, respectively [16]. One a.u. was equal to 100 mL of wine, 330 mL of beer or 50 mL of hard liquor.

### Statistical Analysis

Qualitative data are presented as frequency and relative frequency and were compared using the χ^2^ test. Quantitative data are presented as median and 25th–75th percentiles and tested with Mann–Whitney U test two groups because of non-normal distribution. Distribution was tested using a Kolmogorov–Smirnov test. Spearman’s nonparametric correlation coefficient was used, and MS Excel was used as a database. MedCalc ver. 16.2.1. (MedCalc Software, Ostend, Belgium) was run for statistical data processing. The level of statistical significance was set at 5% (*p* < 0.05) and all confidence intervals were given at a 95% level.

## 3. Results

A total of 58 subjects were included in this study, equally distributed between the OSCC and the healthy control group. None of the subjects had refused to participate or quit during sampling, and all the required information was gathered. Therefore, we report no missing data.

No difference in age (U = 295.00; *p* = 0.051) and sex (χ^2^ = 0.094; *p* = 0.76) between the OSCC and control group was observed. Respondents’ risk habits are shown in Table 1. Respondents’ systemic diseases/conditions and drug consumption are described in Table 2. No statistical difference was found for these relations (data available on request).

**Figure 1:**
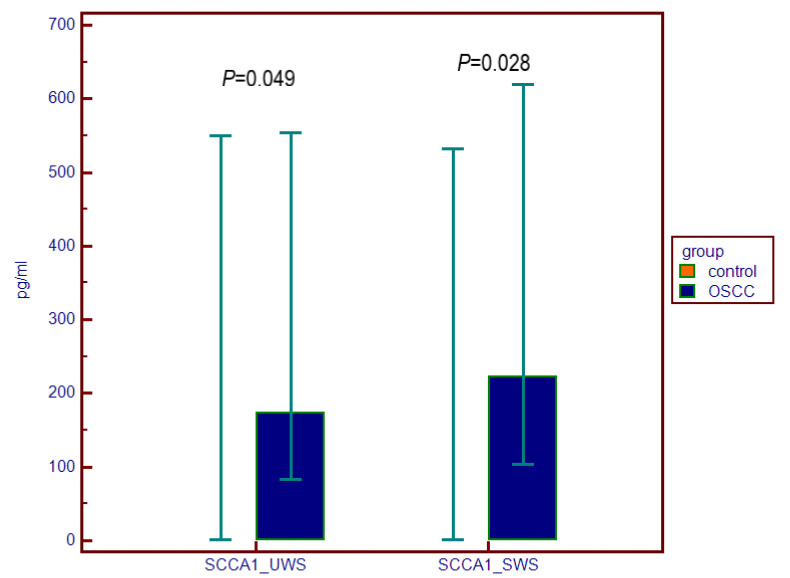
SCCA1 levels (pg/mL) in unstimulated whole saliva (UWS) and stimulated whole saliva (SWS) in patients with oral squamous cell carcinoma (OSCC) and in the control group expressed with median and the 25th–75th percentiles.

**Figure 2:**
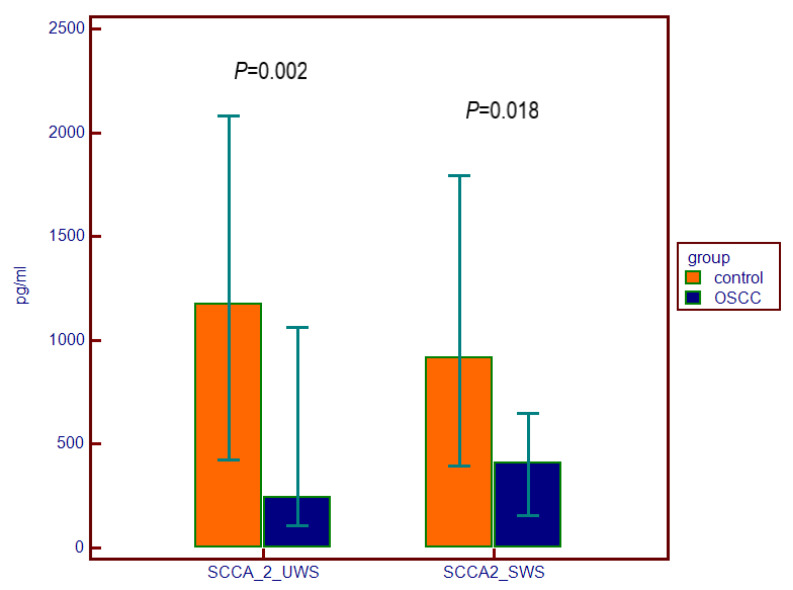
SCCA2 levels (pg/mL) in unstimulated whole saliva (UWS) and stimulated whole saliva (SWS) in patients with oral squamous cell carcinoma (OSCC) and in the control group expressed with median and the 25th–75th percentiles.

Difference between the OSCC group and the control group was found between the levels of SCCA1 and SCCA 2 both in UWS and SWS (Table 3, Figure 1 and Figure 2). TROP2 was not measurable in the majority of salivary samples of both the OSCC and control group and thereby these values have not been processed for statistical analysis.

The most commonly affected location subsite in OSCC patients was the tongue (N = 9; 31.03%), followed by the sublingual region (N = 6; 20.69%), retromolar mandibular gingiva (N = 4; 13.79%), retromolar mandibular region, maxillary gingiva and palate (N = 3 each; 10.34%) and the check (N = 1; 3.45%).

Papilla bleeding index (PBI) was significantly higher in OSCC patients (U = 157.50; *p* < 0.0001).

## 4. Discussion

Squamous cell carcinoma antigens 1 and 2 are both measurable in saliva. The main result of this investigation is the significant difference in SCCA 1 and 2 levels in both UWS and SWS between the patients with early-stage T1N0M0 OSCC and the control group. To our knowledge, this is the first study on salivary SCCA1 and SCCA2 levels in OSCC patients.

Several studies have measured serum SCCA levels in OSCC patients and tried to associate the values with the clinical and pathohistological findings [20,21,22,23,24,25]. A systemic review and meta-analysis by Travassos et al. correlated the high levels of SCCA with TNM stage in head and neck squamous cell carcinomas, lymph node metastasis, tumour depth invasion, recurrence and overall survival [26]. However, head and neck squamous cell carcinomas, although a groups of carcinomas patohistologically similar, should be investigated different entities, due to the different risk factors and different carcinogenesis. Even OSCC with and without metastatic disease should be investigated separately due to the different genomic events and changes occurring during oral carcinogenesis. Therefore, the advantage of this study is the inclusion of only early-stage T1N0M0 OSCC carcinomas.

Derakhshan et al. suggested that the serum levels of SCCA1 are not associated with the expression of this protein in the tissues of squamous cell carcinomas due to the possibility of peripheral T-lymphocyte and tumour cell SCCA1 production [5]. If serum levels and tissue expression of squamous cell carcinoma do not correspond, it is possible that the serum and salivary values also do not.

Apart from squamous cell carcinomas, overexpression of SCCA proteins has been registered also in breast, lung, pancreas adenocarcinomas and hepatocellular carcinomas [27,28,29]. Furthermore, serum SCCA levels were also elevated in atopic dermatitis, psoriasis, asthma and chronic obstructive pulmonary disease [30,31]. Thereby, presence of inflammation could possibly present a confounder. To our knowledge, no information on the levels of SCCA proteins in periodontitis and gingivitis is available in the literature. Levels of SCCA proteins in different squamous cell carcinomas and inflammatory conditions have never been systematically compared.

Both SCCA1 and SCCA2 were measurable in UWS and SWS. However, the SWS has provided slightly better results for both SCCA proteins. It is acknowledged that SWS is composed of mostly parotic saliva, while the UWS mostly of submandibular gland saliva. Saliva from the parotic gland is serous, while saliva from the submandibular gland has both serous and mucous content.

The majority of salivary SCCA1 values in the control group, both in UWS and SWS, were below the limit of detection of the ELISA kit. These results are in line with the allegation that SCCA1 is present in stratified squamous epithelium of healthy individuals but not in the circulation [7].

Interestingly, salivary SCCA1 and SCCA2 levels were inversely proportional in the OSCC group, SCCA1 levels elevated in the OSCC group, and SCCA2 levels lower than in the control group. It would be interesting to investigate this relationship with the OSCC progression in the advanced disease stages. The reason for the low salivary values of SCCA2 in OSCC patients remains unclear.

Several studies separately investigated SCCA 1 and SCCA 2 in different conditions and diseases. A study by Okawa et al. measured the serum SCCA proteins in atopic dermatitis and obtained elevated SCCA1 and SCCA2 in these patients compared to the healthy controls [30]. Psoriasis and allergic diseases can induce both SCCA1 and SCCA2 expression [32]. In peripheral blood mononuclear cells of healthy subjects, SCCA1 is expressed at a higher level than SCCA2 [8]. The same authors mentioned the possibility of the incongruent expression of SCCA1 and SCCA2.

In an immunohistochemical study by Cataltepe et al. strong cytosolic SCCA1 immunoreactivity was registered in the tongue epithelium, while the SCCA2 staining was moderate to low [33]. Interestingly, parotid gland and minor salivary glands were negative for both SCCA proteins and in the submandibular gland duct weak to moderate immunoreactivity in the presence of metaplasia was observed [33]. The same group of authors also investigated the immunoreactivity of SCCA proteins in head and neck and lung carcinomas. Immunoreactivity of SCCA1 and SCCA2 in head and neck squamous cell carcinomas was heterogenous and scattered. In squamous cell carcinomas of the lung, SCCA1 and SCCA2 staining was comparable.

Extremely low levels of TROP2 were obtained in several OSCC patients and control subjects, all of which had levels below the limit of detection of the TROP2 ELISA kit. In other samples, TROP2 was not detected. Therefore, we presume that TROP2 is not measurable in the saliva of T1N0M0 OSCC patients. The possibility of TROP2 presence in saliva in the advanced OSCC stages is questionable.

Altogether, one sample from the OSCC group had higher values than the limit of detection of the ELISA kit for the SCCA2 in the UWS, and two from the control group in the SWS. Using ELISA for salivary samples has its’ well-known drawbacks such as the possible effect of blood contamination, possible influence of plasma molecules from crevicular fluid and the potential high viscosity and thereby poor rheological properties of samples. High viscosity samples were repeatedly centrifugated as described in a paper by Salarić et al. [16].

As expected, the OSCC group smoked more and drank more alcohol. None of the subjects from the control group had drunk alcohol and smoked, i.e., had both most common risk habits for OSCC development.

The respondents had not been controlled by the PBI values, which may present a confounder, since the overexpression of SCCA proteins has been documented in some inflammatory conditions. Patients with OSCC usually have poor oral hygiene, so the higher PBI score obtained in the OSCC group was not unanticipated.

This study has several limitations. To assess the clinical potential of SCCA1 and SCCA2 as OSCC biomarkers, a larger study is needed. HPV infection is usually associated with oropharyngeal carcinoma, but the unknown HPV status could also present a confounding variable, along with the sample size and the absence of a statistical control for risk factors, smoking and alcohol, and PBI. Studies on a greater sample are needed to validate SCCA1 and SCCA2 as clinically valuable biomarkers for OSCC.

## 5. Conclusions

Salivary SCCA1 and SCCA2 are potential biomarkers for T1N0M0 OSCC. Salivary SCCA1 levels were higher in the OSCC group, while the SCCA2 levels were lower in the OSCC group, compared to the values obtained from the control group. TROP2 was not measurable in the saliva of either T1N0M0 OSCC patients or the control group.

## Figures and Tables

**Table 1:** Oral squamous cell carcinoma (OSCC) group and control group risk habits.

	OSCC Group (N = 29)	Control Group (N = 29)	Statistics
Only smoked	3 (10.34%)	1 (3.45%)	/
Only consumed alcohol	6 (20.69%)	0 (0%)	/
Consumed alcohol and smoked	14 (48.28%)	0 (0%)	/
Alcohol altogether	Does not drink: 6 (20.69%) 1 a.u./day: 4 (13.79%) 2–4 a.u./day: 6 (20.69%) >5 a.u./day: 13 (44.83%)	Does not drink: 14 (48.28%) 1 a.u./day: 10 (34.48%) 2–4 a.u./day: 3 (10.34%) >5 a.u./day: 2 (6.90%)	U = 206.00; *p* = 0.0005
Smoking altogether	Does not smoke: 12 (41.38%) 1–5 cig./day: 5 (17.24%) 6–10 cig./day: 0 (0%) 11–20 cig./day: 3 (10.34%) 21–35 cig./day: 8 (27.59%) >36 cig./day: 1 (3.45%)	Does not smoke: 13 (44.83%) 1–5 cig./day: 4 (13.79%) 6–10 cig./day: 6 (20.69%) 11–20 cig./day: 6 (20.69%) 21–35 cig./day: 0 (0%) >36 cig./day: 0 (0%)	U = 349.00; *p* = 0.244
No risk habits	9 (31.03%)	13 (44.83%)	/

OSCC = oral squamous cell carcinoma; SD = standard deviation; a.u. = alcohol unit; cig. = cigarettes.

**Table 2:** Systemic conditions and diseases classified using the International Classification of Diseases 11th Revision (ICD-11) and drug consumption classified using the Anatomical Therapeutic Chemical (ATC) Classification System in the OSCC and control group.

Systemic Condition or Disease (ICD-11)	OSCC Group (N)	Control Group (N)	Total (N)	Drug (ATC)	OSCC Group (N)	Control Group (N)	Total (N)
Healthy/no systemic conditions	/	18	18	Do not take any medication	9	18	27
Hypertension (I10)	12	9	19	Calcium inhibitors (C08)	4	2	6
Anxiety (MB24.3)	7	2	9	Proton pump inhibitors (A02BC)	5	0	5
Type 2 diabetes mellitus (E11)	2	0	0	Acetylsalicylic acid (B01AC30)	3	1	4
Atherosclerosis (I70)	6	2	8	Angiotensin II receptor blockers, plain (C09CA)	2	0	2
Chronic kidney disease (GB61)	1	0	1	Benzodiazepine derivatives (N05CD)	8	2	10
Hyperlipidaemia (5C80Z)	7	6	13	Antidepressants (N06A)	3	1	4
Alcohol-induced psychotic disorder (6C40.6)	1	0	1	Alpha-adrenoreceptor antagonists (C02CA)	5	2	7
Osteoporosis (M81)	1	0	1	Vitamin D and analougs (A11CC)	1	3	4
Depression (F32)	3	1	4	Beta blocking agents, selective (C07AB)	10	1	11
Prostatic hypertrophy (GA90)	4	2	6	Selective beta-2-adrenoreceptor agonists (R03AC)	1	0	1
Asthma (J45)	1	0	1	ACE inhibitors, plain (C09AA)	8	4	12
Gastro-oesophageal reflux disease (DA22)	6	0	6	H2-receptor antagonists (A02BA)	5	0	5
Insomnia (7A00)	2	0	2	HMG CoA reductase inhibitors (C10AA)	7	6	13
				Blood glucose lowering drugs, excl. insulins (A10B)	2	0	2
				Bisphosphonates (M05BA)	1	0	1

OSCC = oral squamous cell carcinoma.

**Table 3:** Salivary SCCA1 and SCCA2 levels comparison between the OSCC and control group.

	SCCA1				SCCA2			
	OSCC Group (N = 29)	Control Group (N = 29)	OSCC Group (N = 29)	Control Group (N = 29)	OSCC Group (N = 29)	Control Group (N = 29)	OSCC Group (N = 29)	Control Group (N = 29)
	UWS		SWS		UWS		SWS	
Minimal value (pg/mL)	46.47	2.00	47.84	2.00	13.51	89.23	17.86	12.25
Maximal value (pg/mL)	1769.36	2497.71	1257.25	2427.18	11,000.00	3245.41	2623.32	11,000.00
Median (95% CI)	173.06 (99.77–471.14)	2.00 (2.00–321.47)	222.79 (111.23–564.69)	2.00 (2.00–207.46)	241.46 (165.79–530.08)	1174.04 (596.67–1922.95	408.40 (201.32–593.50	915.29 (505.01–1736.08)
Interquartile range	82.98–554.12	2.00–550.38	103.64–619.18	2.00–531.87	105.27–1063.51	425.63–2082.20	156.40–646.14	396.58–1794.07
Statistics	U = 295.00	*p* = 0.049	U = 280.00	*p* = 0.028	U = 222.00	*p* = 0.002	U = 268.50	*p* = 0.018

OSCC = oral squamous cell carcinoma; CI = confidence interval; UWS = unstimulated whole saliva; SWS = stimulated whole saliva.

## Data Availability

All data used and/or analyzed during the current study are available from the corresponding authors on reasonable request.

## Supplementary Materials

The following are available online at https://www.mdpi.com/article/10.3390/dj10040070/s1, Supplement 1. Preliminary ELISA experiments and optimization data for SCCA1 and SCCA2 in saliva.
