# Supplementary material for: Salivary Scca1, Scca2 and Trop2 in Oral Cancer Patients—A Cross-Sectional Pilot Study
Source: Dent J (Basel). 2022 Apr 15;10(4):70. doi: 10.3390/dj10040070 (PMC9024948; doi:10.3390/dj10040070)
Supplement: Supplementary file 1 [file dentistry-10-00070-s001.zip › dentistry-1613666-supplementary.pdf]

Supplement 1. Preliminary ELISA experiments and optimization data for SCCA1 and SCCA2 in saliva.

SCCA1 PRE-TESTING

The procedure was carried out according to the manufacturer's instructions, and therefore, a pre-test was performed since the ELISA kits used were not specific for the saliva samples as well as to determine the need for sample dilution in order to keep the samples within the standard curve.

The pre-test revealed low concentrations of SCCA1 in the saliva samples, so control samples (serum and plasma) were used to control the ELISA kit.

SAMPLE DILUTION

The need for diluting samples was analyzed following the ELISA kit manufacturer's instructions.

ELISA PLATE SAMPLE

	1	2	3	4	5	6	7	8	9	10	11	12
A	B	B	1B (1:1)	1B (1:1)	2B (1:1)	2B (1:1)	3B (1:1)	3B (1:1)	5B (1:1)	5B (1:1)	15B (1:1)	15B (1:1)
B	St1	St1	1B (1:2)	1B (1:2)	2B (1:2)	2B (1:2)	3B (1:2)	3B (1:2)	5B (1:2)	5B (1:2)	15SB (1:1)	15SB (1:1)
C	St2	St2	1B (1:10)	1B (1:10)	2B (1:10)	2B (1:10)	3B (1:10)	3B (1:10)	5B (1:10)	5B (1:10)	15SB (1:10)	15SB (1:10)
D	St3	St3	1B (1:25)	1B (1:25)	2B (1:25)	2B (1:25)	3B (1:25)	3B (1:25)	5B (1:25)	5B (1:25)	15SB (1:25)	15SB (1:25)
E	St4	St4	1SB (1:1)	1SB (1:1)	2SB (1:1)	2SB (1:1)	3SB (1:1)	3SB (1:1)	5SB (1:1)	5SB (1:1)	6B (1:1)	6B (1:1)
F	St5	St5	1SB (1:2)	1SB (1:2)	2SB (1:2)	2SB (1:2)	3SB (1:2)	3SB (1:2)	5SB (1:2)	5SB (1:2)	6A (1:25)	6A (1:25)
G	St6	St6	1SB (1:10)	1SB (1:10)	2SB (1:10)	2SB (1:10)	3SB (1:10)	3SB (1:10)	5SB (1:10)	5SB (1:10)	6SB (1:1)	6SB (1:1)
H	St7	St7	1SB (1:25)	1SB (1:25)	2SB (1:25)	2SB (1:25)	3SB (1:25)	3SB (1:25)	5SB (1:25)	5SB (1:25)	6SB (1:25)	6SB (1:25)

St—standard; B and SB—unstimulated and stimulated saliva sample; black—control group; red—oral cancer group

RESULTS

SAMPLE	**CONCENTRATION (pg/ml)**<br>**Second order polynomial 0.9626**
1B (1:1)	16.50174243
1B (1:2)	<
1B (1:10)	<
1B (1:25)	<
1SB (1:1)	<
1SB (1:2)	<
1SB (1:10)	<
1SB (1:25)	<
2B (1:1)	33.51266964
2B (1:2)	<
2B (1:10)	<
2B (1:25)	<
2SB (1:1)	52.86923393
2SB (1:2)	4.820011092
2SB (1:10)	<
2SB (1:25)	<
3B (1:1)	<
3B (1:2)	<
3B (1:10)	<
3B (1:25)	<
3SB (1:1)	<
3SB (1:2)	<
3SB (1:10)	<
3SB (1:25)	<
4B (1:1)	<
4B (1:2)	<
4B (1:10)	<
4B (1:25)	<
4SB (1:1)	<
4SB (1:2)	<
4SB (1:10)	<
4SB (1:25)	<
15B (1:1)	152.8692339333326
15B (1:2)	66.2013313767905
15B (1:10)	18.7661891232367
15B (1:25)	<
15SB (1:1)	266.8989129887211
15SB (1:2)	123.9998202987222
15SB (1:10)	<
15SB (1:25)	<

The suggested results indicated no need for sample dilution.

PREPARATION OF SAMPLES FOR ANALYSIS:

Thawed saliva samples were centrifuged for 20 min at 1000xg; the obtained supernatant was used for analysis (parallel operation; 100 μL sample).

ELISA PLATE sample

	1	2	3	4	5	6	7	8	9	10	11	12
A	B	B	Ser1	Ser1	10B	10B	14B	14B	22B	22B	36B	36B
B	St1	St1	Ser2	Ser2	10SB	10SB	14SB	14SB	22SB	22SB	36SB	36SB
C	St2	St2	Pla1	Pla1	11B	11B	16B	16B	25B	25B	38B	38B
D	St3	St3	Pla2	Pla2	11SB	11SB	16SB	16SB	25SB	25SB	38SB	38SB
E	St4	St4	8B	8B	12B	12B	20B	20B	30B	30B	40B	40B
F	St5	St5	8SB	8SB	12SB	12SB	20SB	20SB	30SB	30SB	40SB	40SB
G	St6	St6	9B	9B	13B	13B	21B	21B	35B	35B	41B	41B
H	St7	St7	9SB	9SB	13SB	13SB	21SB	21SB	35SB	35SB	41SB	41SB

St—standard; Ser—serum sample; Pla—plasma sample; B and SB—unstimulated and stimulated saliva sample; black—control group; red—oral cancer group

Reading of OD (optical density) values was performed at once on a microtiter reader at a wavelength of 450 nm with a differential wavelength of 630 nm.

RESULTS

GraphPad Prism 7.03 personal computer program was used to calculate the results.

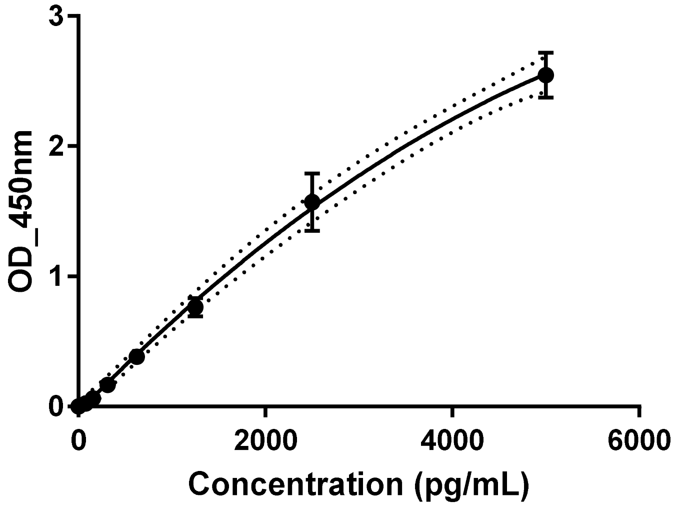

Figure S1. Standard interpolation curve: Second order polynomial (quadratic); R-square: 0.9915.

Interpolation of results with two more standard curves (Third order polynomial - cubic and Sigmoidal, 4PL, X is log concentration) was performed in order to compare the obtained results and obtain the best, most accurate R-square value. The second order polynomial (quadratic) standard curve proved to be the most precise.

SAMPLE	SCCA1 (pg/mL)
Ser1	1712.122398
Ser2	1889.509331
Pla1	1129.110089
Pla2	989.988123
8B	*
8SB	*
9B	*
9SB	*
10B	*
10SB	*
11B	*
11SB	*
12B	*
12SB	*
13B	*
13SB	*
14B	*
14SB	*
16B	*
16SB	*
20B	172.528644
20SB	227.65166
21B	157.432663
21SB	178.712042
22B	417.432663
22SB	477.178671
25B	206.470633
25SB	322.840112
30B	147.840112
30SB	160.861206
35B	364.291137
35SB	562.233012
36B	178.712042
36SB	205.963307
38B	60.175386
38SB	71.841879
40B	112.45686
40SB	104.86973
41B	369.095375
41SB	472.528644

SCCA2 PRE-TESTING

As for SCCA1, the procedure was carried out according to the manufacturer's instructions, and therefore, a pre-test was performed since the ELISA kit was not specific for the saliva samples as well as to determine the need to dilute the samples to keep the samples within the standard curve. Due to their instability, the control of standards was also carried out.

PREPARATION OF SAMPLES FOR ANALYSIS:

Thawed saliva samples were centrifuged for 20 min at 1000xg; the obtained supernatant was used for analysis (parallel operation; 100 μL sample).

ELISA plate sample

	1	2	3	4	5	6	7	8	9	10	11	12
A	B	B	B	B	7C	7C	11C	11C	22C	22C	36C	36C
B	St1	St1	Stt1	Stt1	7SC	7SC	11SC	11SC	22SC	22SC	36SC	36SC
C	St2	St2	Stt2	Stt2	8C	8C	12C	12C	25C	25C	38C	38C
D	St3	St3	Stt3	Stt3	8SC	8SC	12SC	12SC	25SC	25SC	38SC	38SC
E	St4	St4	Stt4	Stt4	9C	9C	20C	20C	30C	30C	40C	40C
F	St5	St5	Stt5	Stt5	9SC	9SC	20SC	20SC	30SC	30SC	40SC	40SC
G	St6	St6	Stt6	Stt6	10C	10C	21C	21C	35C	35C	41C	41C
H	St7	St7	Stt7	Stt7	10SC	10SC	21SC	21SC	35SC	35SC	41SC	41SC

St—standard; C and SC—unstimulated and stimulated saliva sample; black—control group; red—oral cancer group

Reading of OD (optical density) values was performed at once on a microtiter reader at a wavelength of 450 nm with a differential wavelength of 630 nm.

RESULTS

GraphPad Prism 7.03 personal computer program was used to calculate the results.

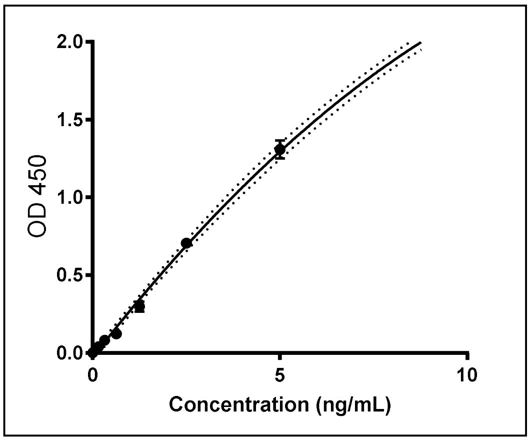

Figure S2. Standard interpolation curve: Second order polynomial (quadratic); R-square: 0,9974.

Interpolation of results with two more standard curves (Third order polynomial - cubic and Sigmoidal, 4PL, X is log concentration) was performed in order to compare the obtained results and obtain the best, most accurate R-square value. The second order polynomial (quadratic) standard curve proved to be most representative.

RESULTS

**SAMPLE**	**SCCA2 (pg/mL)**
7C	448.1798123
7SC	168.0016829
8C	955.9506923
8SC	702.6678509
9C	967.9457857
9SC	514.7789114
10C	246.6476765
10SC	97.85119554
11C	182.7204738
11SC	299.2790458
12C	549.8486883
12SC	748.2792706
20C	1462.205772
20SC	1293.437009
21C	350.4204391
21SC	748.2792706
22C	1309.183976
22SC	405.0301883
25C	208.9182936
25SC	357.0304938
30C	210.5569816
30SC	217.1132942
35C	140.2337296
35SC	491.4394227
36C	243.3635707
36SC	559.8820231
38C	494.7716649
38SC	601.7520143
40C	107.622571
40SC	226.9524511
41C	928.5660983
41SC	145.1307133
